# Can mixed reality technologies teach surgical skills better than traditional methods? A prospective randomised feasibility study

**DOI:** 10.1186/s12909-023-04122-6

**Published:** 2023-03-03

**Authors:** Payal Guha, Jason Lawson, Iona Minty, James Kinross, Guy Martin

**Affiliations:** Department of Surgery and Cancer, Imperial College London, St Mary’s Hospital, 10th Floor QEQM Building, W2 1NY London, UK

**Keywords:** Mixed reality, Clinical competence, Technology assessment, Medical education

## Abstract

**Background:**

Basic surgical skills teaching is often delivered with didactic audio-visual content, and new digital technologies may allow more engaging and effective ways of teaching to be developed. The Microsoft HoloLens 2 (HL2) is a multi-functional mixed reality headset. This prospective feasibility study sought to assess the device as a tool for enhancing technical surgical skills training.

**Methods:**

A prospective randomised feasibility study was conducted. 36 novice medical students were trained to perform a basic arteriotomy and closure using a synthetic model. Participants were randomised to receive a structured surgical skills tutorial via a bespoke mixed reality HL2 tutorial (n = 18), or via a standard video-based tutorial (n = 18). Proficiency scores were assessed by blinded examiners using a validated objective scoring system and participant feedback collected.

**Results:**

The HL2 group showed significantly greater improvement in overall technical proficiency compared to the video group (10.1 vs. 6.89, p = 0.0076), and a greater consistency in skill progression with a significantly narrower range of scores (SD 2.48 vs. 4.03, p = 0.026). Participant feedback showed the HL2 technology to be more interactive and engaging with minimal device related problems experienced.

**Conclusions:**

This study has demonstrated that mixed reality technology may provide a higher quality educational experience, improved skill progression and greater consistency in learning when compared to traditional teaching methodologies for basic surgical skills. Further work is required to refine, translate, and evaluate the scalability and applicability of the technology across a broad range of skills-based disciplines.

**Supplementary Information:**

The online version contains supplementary material available at 10.1186/s12909-023-04122-6.

## Background

Current approaches to surgical skills training commonly utilise the support of didactic video tutorials as these have been shown to effectively teach basic skills such as suturing [1]. However, these techniques may disadvantage kinaesthetic learners and provide limited scope for the development of novel teaching approaches. Simulation and video-based learning is now a corner stone of surgical education, with data suggesting that virtual reality (VR) skills-based learning creates engaging learning environments that promote deeper understanding and long-term retention of knowledge and skills [2], with multi-modal teaching preferred by students [3].

Mixed Reality (MR) technology offers an immersive experience in which real and virtual elements of an environment co-exist. Headsets allow multiple users to remotely link and collaboratively interact through bidirectional communication and interaction with spatially recognised 3D holographic content within the real visualised environment. This technology can therefore provide specific 3D imaging, generic dynamic physiological and anatomical models, or procedural animations to improve the learning offer, whilst also allowing remote access to educations that may widen accessibility and lower barriers to educational opportunities. The HoloLens 2 (HL2) (Microsoft Corporation, Redmond, WA, USA) is an untethered MR headset, and is an example of such technology. It has been successfully used across a range of educational settings including teaching ward rounds [4], and human anatomy instruction through bespoke 3D interactive models that have been shown to give a better understanding of anatomical structures compared to standard lecture-based teaching [5–8]. In addition, the technology has been demonstrated to be a potential method to teach basic practical skills such as digital rectal examinations and urinary catheterisation [9, 10]. The use of augmented (AR) and virtual reality (VR) for surgical training has been widely reported [11, 12], but there remains a paucity of evidence for the potential effectiveness of newer MR technology to support the delivery of basic surgical skills training. MR technology has the potential to enhance all aspects of skills training, and specifically self-directed, remote, or large-group teaching where resource constraints do not allow for instructor-led face-to-face tuition and video-based methods are currently the mainstay of delivery. This study therefore sought to examine the impact of MR technology on basic surgical skills training in a novice cohort when compared to traditional video-based methods of teaching.

## Methods

### Participants

36 novice clinical medical student participants were recruited. All had undergone summative assessment in simple suturing competencies as part of their curriculum prior to participation in the study but had not previously undertaken the surgical skill being taught and assessed. Demographic data were collected, and preferred learning style data self-reported by participants using the VARK (visual, auditory, read/write, kinaesthetic) model; a validated inventory that identifies student learning preferences [13].

### Study Design

This was a single blind randomised study with 18 participants randomised to each group. Each group received a structured surgical skills tutorial on performing and closing an arteriotomy: a conventional video-based tutorial group, and a MR HL2 group. The allocation ratio for each group was 1:1 via block randomisation prior to participation. The study received institutional educational ethics approval (EERP2021-027a) and informed consent was obtained from all participants.

**Fig. 1:**
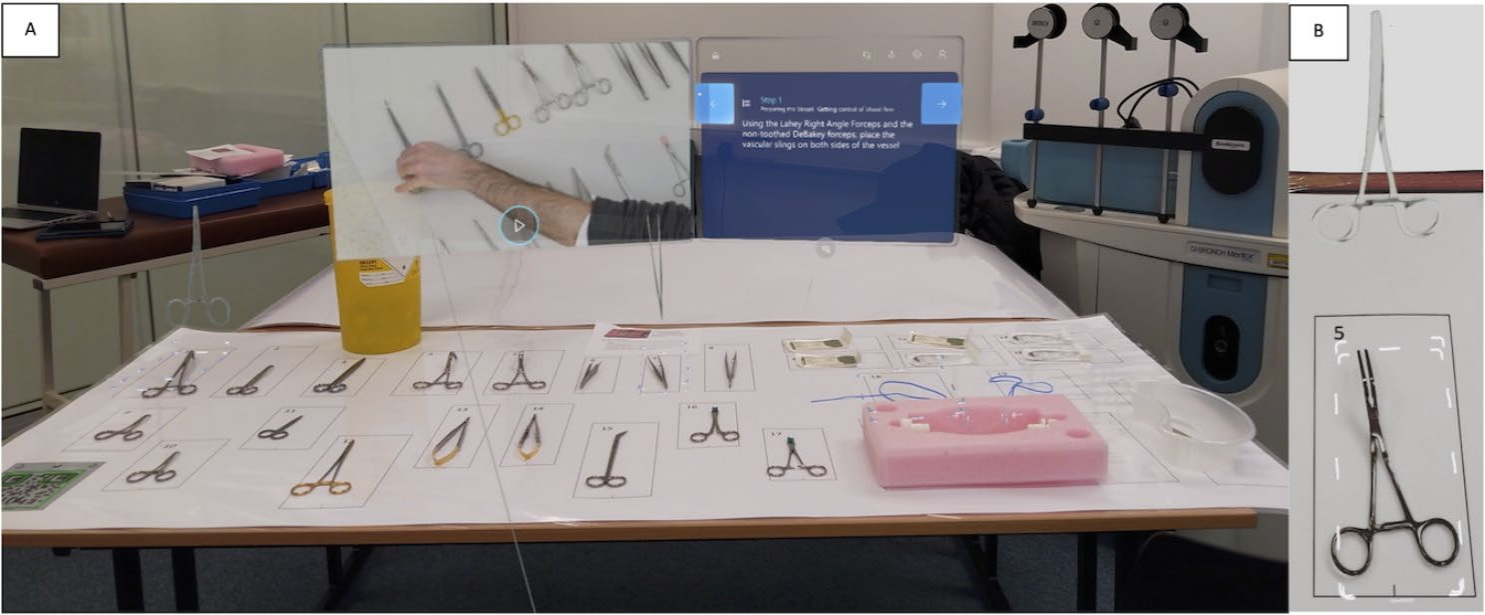
Screenshot of the HoloLens teaching session content. The instructions are displayed on a screen in front of the participant whilst an embedded video clip of the step is shown to the left of the instructions. Instruments are highlighted with a 3D hologram in turn, and the surgical model is labelled to identify correct clamp placement (1 A). Close up image demonstrating how the technology highlights the correct instrument in order of use for the student (1B)

The study took place in three stages and employed a modified Peyton’s four-step approach to skills teaching for both groups [14]. Participants undertook a baseline assessment of surgical proficiency, received a standardised surgical skills tutorial, and then performed a further assessed task to determine skill progression. Participants were trained to perform a simple arteriotomy and closure on a commercial bench top synthetic surgical model (Limbs & Things Ltd, Bristol, UK). They performed a longitudinal arteriotomy that was then closed with interrupted sutures. A description of the scenario can be found in *Appendix A*. In the first stage, all participants were provided with identical basic written instruction on how to undertake an arteriotomy and closure. The task was then performed on a surgical model to provide a baseline assessment of proficiency. In the second stage, the video group received tuition through a pre-recorded structured skills video. Participants were able to independently replay all, or parts of the video at their preferred pace while practising the skill for 20 min. The HL2 group received instruction on how to fit and use the HL2 device and technical support was provided for participants who had difficulties in using the technology. They received the same structured surgical tutorial that was enhanced with the addition of bespoke interactive holographic content and instruction delivered through Microsoft Dynamics 365 Guides (Microsoft Corporation, Redmond, WA, USA), and were again given 20 min to practice the skill whilst utilising the MR content. The MR tutorial provided to the HL2 group is shown in Fig. 1, and provided simultaneous and interactive written instruction, together with embedded video of each procedural step and live instrument identification and use guidance. In the final stage both groups then undertook a further task assessment to provide an objective assessment of skill progression. Once all tasks had been completed, participants were able to experience the alternative teaching modality to ensure no participant was disadvantaged and all had exposure to both interventions. Participant demographics, learning style information, and teaching and device feedback were collected using 5- point Likert style scale responses scored 1–5 with 1 being “strongly disagree” and 5 being “strongly agree.”

Assessment tools such as the Objective Structured Assessment of Technical Skill (OSATS) have been shown to measure surgical skill proficiency accurately and validly [15]. In this study proficiency was measured by evaulating correct instrument selection, task stage completion, and suture quality to produce an overall proficiency score. The surgical skill was divided into several stages, and a modified task specific OSATS checklist was developed to assess corresponding instument selection and task stage completion. This was completed by an examiner, blinided to the participants instructional condition, during each assessed task. The time taken to complete the task was also recorded with a cut-off of 15 min allowed. A second blinded expert reviewer then assessed each completed arteriotomy model to assess suture quality and error using a task-specific score. An overall proficiency score, based on a metric-based performance score with evidence for its validity [16, 17], was then calculated by combing instrument selection and suture quality scores to determine overall skill progression on a per-participant basis. The surgical proficiency scoring system is provided in *Appendix B*.

### Statistical analysis

Standard descriptive statistics were utilised. The distribution of data was checked for normality using the Shapiro-Wilks test. Comparison of scores between groups were analysed by a T-Test for parametric data and a Mann-Whitney U (MWU) test for non-parametric data. Variance was assessed by an F-test. A Chi-squared test was used to test relationships between categorical data. P-values < 0.05 were considered significant. Statistical analysis was carried out using GraphPad Prism for Mac Version 9.0.0. Data is displayed as mean ± standard deviation.

## Results

### Participant characteristics

There was no difference in age (22.4 years ± 2.45 vs. 22.6 ± 2.52, p = 0.735) or sex (7 vs. 8 male, p = 0.735) between the HL2 and video groups respectively.

### Overall proficiency score

**Fig. 2:**
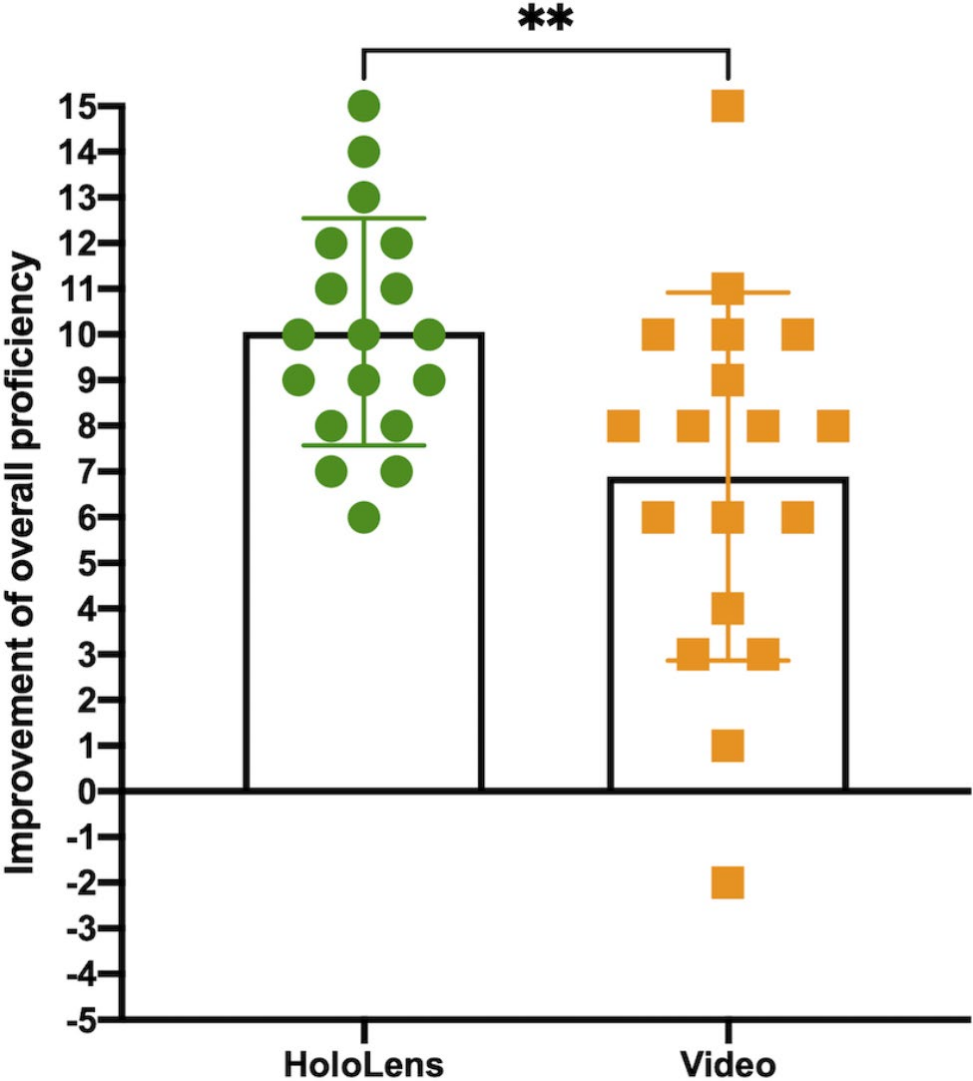
Bar chart depicting the mean proficiency score gain of each group (n = 18/group) on a per participant basis. Those in the HoloLens group showed a significantly greater raw point improvement in proficiency scores (10.1 ± 2.48 vs. 6.89 ± 4.03, p = 0.0076) and greater consistency in skill progression (SD 2.48 vs. 4.03, F_17,17_ = 2.64 p = 0.026)

There was no significant difference in total baseline proficiency scores for the HL2 and video groups (9.11 ± 3.36 vs. 10.2 ± 2.66, p = 0.303). The HL2 teaching group showed a significantly greater gain in raw point proficiency scores compared to the video group (10.1 ± 2.48 vs. 6.89 ± 4.03, p = 0.0076) as illustrated in Fig. 2. The HL2 teaching group also displayed a narrower range of scores, indicating a greater level of consistency in skill progression compared to the video group (SD 2.48 vs. 4.03, F_17,17_ = 2.64 p = 0.026).

### Instrument selection

There was no significant difference in baseline scores for instrument selection for the HL2 and video groups (3.06 ± 0.873 vs. 3.61 ± 1.42, p = 0.246). The HL2 teaching group subsequently had a significantly higher mean score for instrument selection choice when compared to the video group (9.67 ± 0.767 vs. 6.67 ± 1.97, p < 0.0001).

### Suture quality

There was no significant difference in baseline scores for suture quality for the HL2 and video groups (7.17 ± 2.83 vs. 7.50 ± 2.28, p = 0.700), and both groups demonstrated equal improvement in performance (2.22 ± 2.13 vs. 1.94 ± 3.21, p = 0.761).

### Participant learning style and performance

**Fig. 3:**
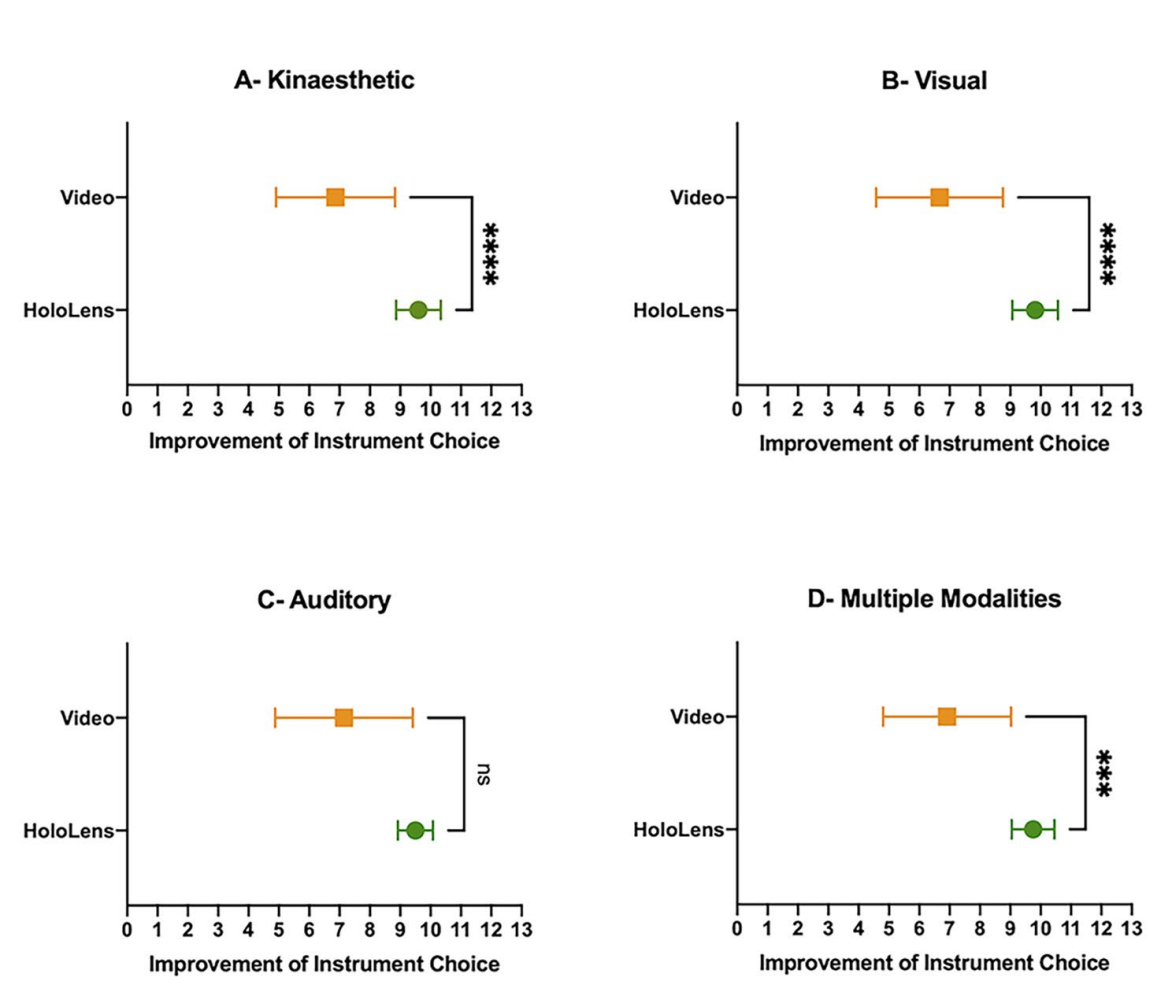
Charts depicting the mean gain in procedural knowledge through instrument selection choice in participants who selected (A) kinaesthetic (n = 30 - HL2 15, video 15), (B) visual (n = 26 - HL2 11, video 15), (C) auditory (n = 11 - HL2 4, video 7) and (D) multiple modalities (n = 20 - HL2 12, video 8) as preferred learning modalities. * = p < 0.05, ** = p < 0.01, *** = p < 0.001, ****=p < 0.0001

The impact of participant learning styles on proficiency are summarised in Fig. 3 Overall, 20 (56%) participants reported a multi-modal learning style, with the remaining 16 (44%) participants reporting a single preferred learning style. Participants who listed ‘kinaesthetic’ (n = 30 - HL2 15, video 15) as one of their learning modalities showed significantly improved scores for instrument selection in the HL2 group (9.60 ± 0.737 vs. 6.87 ± 1.96, p < 0.0001). Improvements in instrument selection scores were also significantly better in the HL2 group for participants who included ‘visual’ (n = 26 - HL2 11, video 15) as one of their preferred learning styles (9.82 ± 0.751 vs. 6.67 ± 2.09, p < 0.0001). However, there was no significant difference in instrument selection scores for participants who listed “auditory” (n = 11 - HL2 4, video 7) as one of their learning modalities (9.50 ± 0.577 vs. 14 ± 2.27, p = 0.112). Multi-modal participants (n = 20 - HL2 8, video 12) who listed more than one modality of learning also showed significantly greater gains in instrument selection scores in the HL2 group compared to the video group (9.75 ± 0.707 vs. 6.92 ± 2.11, p = 0.0009). No difference in suture quality scores between the two study groups across different learning styles was observed.

### Participant feedback

Participant feedback is summarised in Table 1. A minority (6/36, 16.7%) of students felt that surgical skills are currently well taught at their institution. All participants were positive about both teaching modalities and found them easy to use. Teaching using MR content via the HL2 was thought to be more effective than traditional video tutorials (4.50 ± 0.618 vs. 3.83 ± 0.924, p = 0.024), and confidence in performing the skill assessed was better, although not significantly in participants in the HL2 group (2.47 ± 0.624 vs. 1.94 ± 0.802, p = 0.0556). Most participants reported no difficulties or problems with either teaching modality (HL2 26/36, 72.2%. Video 24/36, 66.7%). A minority of participants in the HL2 group reported symptoms or difficulties with wearing the device, with headache reported by 11.1% (2/18) and fatigue or difficulty concentrating by 16.7% (3/18). However, similar difficulties were also reported in the video group with 16.7% (3/18) reporting difficulty concentrating or fatigue.

**Table 1:** Summary of participant feedback (n = 36) scored on Likert scale responses (1–5, not confident / strongly disagree - most confident / strongly agree) or via yes/no responses

	HoloLens	Video	p-value
Procedural confidence	2.47 ± 0.624	1.94 ± 0.802	0.0556
Effectiveness of programme	4.50 ± 0.618	3.83 ± 0.924	0.0242
Ease of use	4.28 ± 0.669	4.17 ± 0.707	0.745
Enjoyment	4.56 ± 0.511	4.06 ± 0.873	0.0821
No symptoms	13	12	0.718
Difficulty concentrating	0	3	0.0704
Headache	2	0	0.146
General discomfort, fatigue, difficulty concentrating	1	1	> 0.999
General discomfort, fatigue	2	1	0.547
Sore eyes	0	1	0.311

## Discussion

This randomised study has shown that students who participate in surgical skills tuition delivered via MR technology demonstrate greater and more consistent skill progression, in addition to reporting a higher quality educational experience when compared to traditional video-based skills tuition.

The HoloLens teaching session gave consistently better results to all learners. Specifically, it led to significant improvements in instrument selection and more consistent improvements in technical performance. These observations could be due to the nature of the HoloLens content that supported a more structured approach to skill practice for the participant. The interactive tutorial provided reinforcement and immediate confirmation of the key procedural steps by producing holographic representations of each surgical instrument in order of use, in addition to highlighting the correct instrument within the surgical field and providing visual guidance for exact instrument placement on the surgical jig, as illustrated in Fig. 1. In accordance with pedagogical best practices, this material was presented to the student in a guided and segmented fashion, with progression through the tutorial via hands-free control to allow content manipulation whilst simultaneously performing the task. Conversely, the video group was only able to practice and re-watch the video content using a self-directed approach with no temporal relationship between the video and task. In addition, they had to put down their instruments and disengage from performing the task to control the video that was presented on an adjoining computer. The interactive learning and enhanced feedback for the HoloLens group likely account for higher instrument selection scores and greater consistency of learning for this group.

Differing participant learning styles appeared to influence the effectiveness of the intervention. Participants were either multi-modal learners or reported a single preferred learning style, with an equal distribution across each study group. Participants who included auditory learning as one of their preferred learning styles performed similarly across the two study groups as would be expected given that the audio commentary provided for both teaching modalities were identical. Participants who included visual and kinaesthetic learning as one of their preferred learning styles performed significantly better in the HoloLens group. Although both groups received the same video material and practice time, the addition of holographic content and markers, and interactive task guide in the HoloLens group created a more dynamic learning environment that supported a multi-modal approach to learning. It is well documented that more interactive teaching sessions delivered through a diverse range of media lead to increased engagement and better performances [18, 19].

Suture quality remained consistent across the two interventions. This was a secondary measure of the study, and instructions and tuition on improving suture quality were not delivered with novel MR content. The lack of significant difference between groups may be accounted for by the limited specificity of the suture quality assessment methodology, however, this observation may also reflect the fact that the students did not have the opportunity to reach a performance plateau in their motor skills [20], with insufficient repetitive experiential learning to yield significant improvements in technical quality. However, the HoloLens could readily address this short coming and future models could visualise correct knot quality measures, by superimposing an animated hologram over the participant’s hands showing the correct technique, allowing the participant to mirror the movements [21]. This would provide a more interactive way to demonstrate and give immediate confirmation of technique for aspects such as correct positioning and tying techniques.

The global need for improved surgical skills teaching is clear [22], and this has been exacerbated during the COVID crisis which has placed significant strain on surgical training. These data suggest that MR technology may have a role to play in addressing these challenges, and that this it is acceptable to students. All HoloLens participants reported enjoying the teaching session, but this observation was not found in the video group. Reasons for a lower enjoyment in the video group can be seen in the written feedback where participants said it was not “interactive” enough and re-watching the video while practising was “too difficult” as they were unable to effectively focus on the task at hand whilst simultaneously controlling and engaging with a non-segmented and poorly accessible passive video. This is in keeping with previous studies that suggest interactive and active learning styles perform better than traditional teaching methods [23, 24]. However, this may represent a bias towards the MR technology amongst participants due to its novelty or inclusion in the study. Nonetheless, high student satisfaction is crucial with educational programmes as it encourages improved engagement and better overall performance [25–27].

Unfamiliarity and the learning curve associated with new technologies often results in slow adoption [28], however, it is promising that no HoloLens participant disagreed with the statement that the technology was easy to use. There is however a learning curve to the technology, and some described that it was initially difficult to interact with some components, but this was rapidly over come with the standardised onboarding protocol. Surprisingly, not all video participants selected “strongly agree” for ease of use for watching the videos. The feedback given suggests this was due to the need to scroll through the video in the practice round which may be challenging whilst simultaneously attempting to practice the technique. This problem is mitigated with the HoloLens teaching programme as Microsoft Dynamics 365 Guides uses a cursor controlled by head movement and gaze. Therefore, the participant did not need to remove their hand from any instruments to continue to the next step or repeat the most recent instruction. This is particularly important when it comes to surgical training as the programme doesn’t disrupt the flow of practice and allows muscle memory to form, leading to enhanced performance of the skills [29]. An interesting observation would be that both groups had increased confidence after the teaching session, however this was slightly higher in the HoloLens group. This could be due to the immediate confirmation of the selected instrument and guidance for its correct placement on that surgical jig that the HoloLens tutorial provided; functionality that is not provided in video-based tuition. Many of the instruments used had not been seen by this novice group before, and therefore such positive identification, feedback and reinforcement aids in learning and correctly identifying the differences between similar-looking instruments.

Most participants (72.2%, 13/18) who used the HoloLens did not experience any negative symptoms with the device, with the few symptoms that were experienced being mild. Studies have shown that prolonged use of the HoloLens may lead to symptoms such as headaches or fatigue [30]. The video group had a similar number of participants with no symptoms, with the most notable symptom reported being difficulty concentrating. This is emphasised by the feedback given that the videos were “long” and “boring”. This was surprising as both groups were provided with the same basic video content, although delivered via very different and contrasting methodologies, but only one participant from the HoloLens group felt like they had difficulty concentrating likely due to the segmented nature of the video content and additional interactive functionality they experienced. These results are encouraging as they show that it is possible to incorporate the interactive nature of MR learning without the major side effects such as “cybersickness” [8, 31].

A key limitation of this study was provided by the technology itself which remains in relative infancy. The battery life of the device and other technical aspects of its operation were principal concerns. When running a Microsoft Dynamics 365 Guide, such as used in this study, it is much shorter than advertised and lasts for only 90 min. Whilst not of direct consequence in this study as each participant used the device for 40 min, it may limit the scalability of the technology across a wider range of subject areas and skills that require a longer instructional period. Further to this, periods of minimal head movement, for example whilst watching a video through the device, can lead it to automatically go to sleep and therefore interrupt the tutorial. Finally, the device is designed to be operated through specific hand gestures such as tapping the inside of the wrist with two fingers to return to the main menu. Whilst through to be unique in other settings, these were sometimes confused with normal movements undertaken in clinical contexts such as putting on gloves which once again may unintentionally disrupt the tutorial; this could be easily resolved through basic software developments that consider the clinical context. This study was conducted in an institution that had the technical resource to develop the bespoke content, and financial resource to purchase the devices which cost of around $3,500 each. Expert technical knowledge is required to effectively create and deliver the MR content, even with a mature software package such as Dynamics 365 Guides that was used in this instance. A multi-step process is required to film and segment the video content, generate bespoke 3D holographic content through a distinct software application, create a task-specific spatially orientated practice environment in which the suturing skill was performed and finally to link these in a single joined-up interactive guide. This resource requirement may act as a barrier to adoption and the potential scalability of the technology, however, over time it is likely the cost of the technology will fall and therefore it will become a more feasible option for widespread use in medical education. In addition, any technology-based trial, particularly involving novel technology, will likely be affected by some degree of technology bias.

Further to the technical limitations, this study looked solely at improvements in proficiency in a tightly defined individual task over a short period of time, potentially limiting the applicability of the findings to generalised surgical proficiency over the longer term. Secondly, whilst insignificant, there was a difference in baseline participant performance between each group that may have partially contributed to the observed findings. Future studies would benefit from retesting participants at longer time interval to determine if MR teaching methods minimise longitudinal skill fade and improve knowledge retention, and from stratifying participants according to baseline proficiency to assess their effectiveness across different performance levels. It is also important to assess the efficacy of the technology across a wider range of topics, skills and contexts.

## Conclusion

This study has shown that a HoloLens delivered mixed reality teaching programme has the potential to produce greater skill progression, more consistency in learning and a higher quality educational experience compared to traditional video-based methods for surgical skills training. Although in its infancy, further work to refine, translate and evaluate the scalability and applicability of the technology will allow it to be readily adopted across a broad range of skills-based disciplines.

## Electronic supplementary material

Below is the link to the electronic supplementary material.

Supplementary Material 1

Supplementary Material 2

## Data Availability

The data generated and/or analysed during the current study are not publicly available as they contain potentially identifiable participant information. They are available from the corresponding author on reasonable request.
