# Supplementary material for: Can mixed reality technologies teach surgical skills better than traditional methods? A prospective randomised feasibility study
Source: BMC Med Educ. 2023 Mar 3;23:144. doi: 10.1186/s12909-023-04122-6 (PMC9985210; doi:10.1186/s12909-023-04122-6)
Supplement: Supplementary file 1 — Supplementary Material 1 [file 12909_2023_4122_MOESM1_ESM.docx]

**Appendix A: Arteriotomy Scenario / Procedure**

**
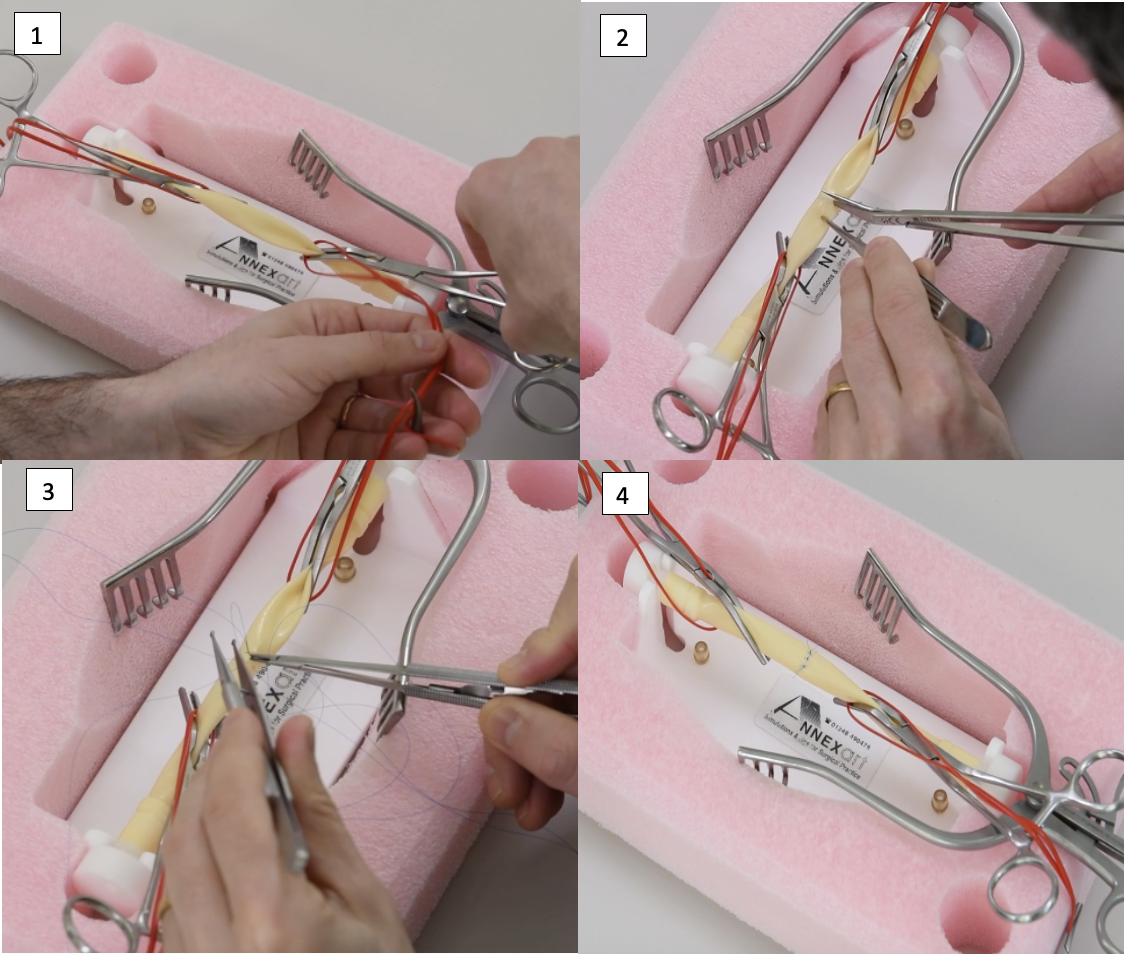
**

1. **Vessel preparation**
   1. Control the vessel with slings
   2. Apply clamps
2. **Arteriotomy**
   1. Perform a transverse arteriotomy
3. **Arteriotomy repair**
   1. Place 3x interrupted non-absorbable monofilament sutures
   2. Tie each suture in turn
   3. Ensure adequate eversion, tension and alignment of the repair
4. **Completion**
   1. Assess adequacy of repair
   2. Remove the clamps and slings
