# Supplementary material for: Can mixed reality technologies teach surgical skills better than traditional methods? A prospective randomised feasibility study
Source: BMC Med Educ. 2023 Mar 3;23:144. doi: 10.1186/s12909-023-04122-6 (PMC9985210; doi:10.1186/s12909-023-04122-6)
Supplement: Supplementary file 2 — Supplementary Material 2 [file 12909_2023_4122_MOESM2_ESM.docx]

**Appendix B: Surgical Proficiency Score (total score /24)**

**Suture Quality Scoring**

Is the incision size appropriate? Y/N?

Was the arteriotomy done vertically across the vessel? Y/N?

Did the Participant use the 5.0 prolene suture? Y/N?

How many sutures were completed? 0,1,2,3

Are the sutures appropriately spaced? Y/N?

Are the sutures appropriately aligned? Y/N?

Are there an appropriate number of knot throws? Y/N?

Did the sutures have the correct tension? Y/N?

Were the sutures cut to an appropriate length? Y/N?

Are the bite sizes appropriate? Y/N?

Are the edges everted? Y/N?

**Instrument Selection Scoring**

Did the Participant use the non-toothed DeBakey forceps? Y/N?

Did the Participant appropriately place the slings? Y/N?

Did the Participant use the Halstead Curved Mosquito? Y/N?

Did the Participant place Angled DeBakey arterial clamps distal to the vascular slings? Y/N?

Was a blade use to make the initial incision? Y/N?

Did the Participant use the potts scissors to extend the incision? Y/N?

Was the arteriotomy done vertically across the vessel? Y/N?

Did the Participant use the 5.0 prolene suture? Y/N?

Was a new prolene suture used for each suture? Y/N?

Did the Participant use the Ratcheted Castroviejo to hold the suture needle? Y/N?

Did the Participant hold the two suture edges in place with a Rubber shod clamp? Y/N?

Did the Participant cut off the sutures with the mayo scissors? Y/N?

How long did the participant take to complete the arteriotomy?
